# Indices of Narrative Language Associated with Disability

**DOI:** 10.3390/children10111815

**Published:** 2023-11-15

**Authors:** Norah M. Almubark, Gabriela Silva-Maceda, Matthew E. Foster, Trina D. Spencer

**Affiliations:** 1 Department of Communication Sciences and Disorders, College of Behavioral and Community Sciences, University of South Florida, Tampa, FL 33612, USA; 2 School of Psychology, Universidad Autonoma de San Luis Potosi, San Luis Potosí 78399, Mexico; gabriela.silva@uaslp.mx; 3 Rightpath Research & Innovation Center, College of Behavioral and Community Sciences, University of South Florida, Tampa, FL 33612, USA; mefoster@usf.edu; 4 Department of Applied Behavioral Science, University of Kansas, Lawrence, KS 66045, USA; trinaspencer@ku.edu

**Keywords:** academic language, language sampling, narrative discourse, disability

## Abstract

Narratives skills are associated with long-term academic and social benefits. While students with disabilities often struggle to produce complete and complex narratives, it remains unclear which aspects of narrative language are most indicative of disability. In this study, we examined the association between a variety of narrative contents and form indices and disability. Methodology involved drawing 50 K-3 students with Individual Education Programs (IEP) and reported language concerns from a large diverse sample (*n* = 1074). Fifty typically developing (TD) students were matched to the former group using propensity score matching based on their age, gender, grade, mother’s education, and ethnicity. Narrative retells and generated language samples were collected and scored for Narrative Discourse and Sentence Complexity using a narrative scoring rubric. In addition, the number of different words (NDW), subordination index (SI), and percentage of grammatical errors (%GE) were calculated using computer software. Results of the Mixed effect model revealed that only Narrative Discourse had a significant effect on disability, with no significant effect revealed for Sentence Complexity, %GE, SI, and NDW. Additionally, Narrative Discourse emerged as the sole significant predictor of disability. At each grade, there were performance gaps between groups in the Narrative Discourse, Language Complexity, and SI. Findings suggest that difficulty in Narrative Discourse is the most consistent predictor of disability.

## 1. Introduction

Nearly 52% of students who receive special education services are classified under specific learning disabilities or speech or language impairments [1]. Many of the other school-based disability categories also involve difficulty with language or communication. One such disability, Developmental Language Disorder (DLD) is a neurodevelopmental condition that appears in childhood and persists throughout a person’s life, characterized by difficulties in learning, understanding, and/or using spoken language without an association with other disorders [2,3]. Students with DLD often exhibit a variety of communication difficulties that adversely impact their social interactions and academic performance. Hence, students with DLD must be identified early to receive the necessary individualized education support and intensive instruction to improve their academic and social achievement [4,5].

### 1.1. Methods for Identifying Language Disabilities 

To identify students with language-related disabilities, SLPs rely heavily on norm-referenced tests (NRTs) [6]. NRTs are typically administered using standardized materials, tasks, and procedures under highly controlled conditions that require a child to engage in behaviors that simulate language but are not necessarily the discourse-level language used in authentic contexts. Despite their efficiency and utility for diagnosing disabilities, NRTs are not infallible. For example, kindergartners may perform at their grade level on an NRT despite having weak syntactic complexity and narrative discourse ability [7], which can result in the misidentification of students who would benefit from early support. Due to the limitations of NRTs, language sampling is often recommended as an alternative or supplement to NRTs [8,9]. It is no surprise that the American Speech-Language-Hearing Association (ASHA) considers language sampling to be an essential part of the speech-language pathologists’ (SLPs) assessment process [10].

Language sampling involves the audio (or video) collection of a child’s language. Once a sample has been transcribed, it can be analyzed for specific language features of interest [11]. The inclusion of a language sample when determining disability has many clinical benefits. SLPs can examine a child’s language as it is used in a meaningful context. Typical language sampling tasks such as play or storytelling have similar processing demands to what students encounter in everyday routines [12]. Because language sampling can occur in contexts in which spontaneous language is generated, it has superior ecological validity, which makes it useful for informing intervention and goal development [13,14,15,16,17]. Language sampling is also a sensitive method for identifying language disabilities across age groups and cultures [13,14,18,19]. Therefore, the current best practice for SLPs is to augment the information obtained from NRTs, when their use is mandated, with a more culturally relevant and authentic assessment of students’ oral language use in meaningful contexts such as language sampling [2,13,20].

### 1.2. Narrative Language Sampling and Analysis

Language can be sampled in different contexts that include conversation and expository and narrative registers. Researchers have known for years that narrative language has a uniquely powerful influence on many academic repertoires. This is because narratives are constructed from the complex literate language that is needed in academic settings. In fact, narratives are commonly used to elicit the complex language of school-aged students [21,22,23,24]. Additionally, narratives are common tools for social engagement. For example, students tell stories about their daily interactions and experiences. Because of their academic and social relevance [25,26], narratives are suitable for language sampling.

Narrative language sampling allows clinicians to capture a snapshot of a child’s true linguistic ability, but the manner in which it is quantified, coded, or measured has the greatest impact on the decisions that can be made [9,15,27]. Regarding the constructs of what is measured, narrative samples are often conceptualized according to their content (named “macrostructure” in some studies), and their form (“microstructure”). At the macro level, story grammar refers to the rules for ordering and grouping the narrative content, and this variable is usually characterized by the inclusion and clarity of story grammar elements. This is more indicative of the narrative content. At the micro level, the complexity of the sentences and the novelty of the words used to tell or retell the story are considered [28,29,30,31]. The examination of narrative language at the word and sentence level aligns more closely with the form of language. Both content and form can be quantified in the same language sample and both types of analysis contribute to the overall story quality [32]. However, the time and skill required to analyze both may be unreasonable for busy school-based SLPs. Therefore, it is prudent to explore the extent to which content and form variables are useful and/or necessary. 

Because measures of content and form reflect linguistic proficiency, they have been used to differentiate students with language disabilities from students with typical language development [31,33]. As the language production of high-quality narratives demands discourse-level content knowledge, linguistic knowledge, and word knowledge [34,35,36], we expect students with disabilities to produce narratives of a reduced quality with respect to content and form indices. This is reflected in the research on the narrative language performance of students with and without disabilities. 

### 1.3. Indices of Narrative Form and Their Relation to Disability

There is a large corpus of research suggesting that narrative form is useful for identifying language disability [37,38]. Narrative language form is most often ascertained by quantifying a child’s inclusion of the grammatical features of complex language [28,30,31,39]. The commonly reported measures in the literature include indices for grammaticality, lexical diversity, and syntactic complexity [40,41]. The evidence for each of these indices for identifying disability is described in the sections below. To ensure consistency in our reviews, we have used the term DLD to refer to participants with language disabilities, even though they may have been originally described as having language impairment (LI), specific language impairment (SLI), or DLD in the original studies. We acknowledge the challenge associated with merging these terms, but the extensive and still debated criteria for each of the terms go beyond the scope of this research. 

#### 1.3.1. Grammaticality

Grammaticality is a measure of grammatical errors in each C-unit or T-unit (e.g., ungrammatical verb forms, ungrammatical pronouns, and ungrammatical morphemes). There is some consensus that grammatical errors are a persistent problem in school-aged students with DLD, as reported in multiple studies. The proportion of grammatical errors in C-units has been found to be significantly different in DLD children compared to their typically developing peers in the second [42,43,44] and fourth grades [42]. However, the differences between studies are considerable. Focusing only on the directly comparable age group within second grade, ref. [44] documented a 49% error rate difference between children with and without DLD, while ref. [43] reported a 23% difference, and ref. [42] found only an 8% difference for the same age group. It is well documented that grammatical errors in the narratives of students with DLD do not disappear. Rather, weaknesses in narrative production continue, and possibly worsen, as language demands increase [45].

For older children, between the ages of 9 and 12 years old, and the proportion of grammatical errors per T-Units, ref. [46] found a 41% error rate difference between 20 students with and 20 without DLD. Similarly, ref. [33] found a 24% error rate difference between 11-year-olds with and without DLD. 

#### 1.3.2. Lexical Diversity

Even when other measures exist, the Number of Different Words (NDW) is the measure that has been used consistently in the literature to evaluate differences in lexical diversity in narrative production between students with and without disabilities, and it is routinely computed using automatic language analysis software such as Systematic Analysis of Language Transcripts (SALT) 20 software [47]. There is also converging evidence that NDW is a reliable indicator for the differentiating of students with and without language disorders up to fourth grade. Ref. [42] found that students with DLD in second and fourth grade generated narratives with a significantly reduced NDW, compared to the TD grade-level matched students. Similarly, ref. [48] found that the NDW from narrative language samples collected from 77 students with DLD between the ages of 4 and 9 years old was lower (85.1 NDW at age 4 and 161.2 NDW at age 9) than the NDW produced by 300 typically developing students (127.7 NDW and 169.9 NDW at the same ages, respectively). The NDW group differences appear to shrink with age, which is consistent with [33] finding that 11-year-old children with DLD and their age-matched controls do not differ significantly in NDW production (56.1 and 60.6 NDW, respectively) [48].

#### 1.3.3. Syntactic Complexity

Syntactic complexity has been measured in several different ways. In some studies, they have used the Subordination Index (SI), or clausal density, which is a measure of the number of clauses in each C-unit, an independent clause with its modifiers [49]. Others have used the proportion of complex coding units (C- or T-units) in relation to the total number of coding units.

Of the studies cited above, some did not find significant differences in SI between children with and without DLD in the second and fourth grades [42], nor in 11-year-olds [33]. Meanwhile, in other studies, significant differences among 7- to 10-year-olds were found [44]. Other researchers using the proportion of complex coding units (C- or T-units) have found that the proportion of complex (and correct) T-units predicted DLD in children aged 9 to 12 years [46]. In the same developmental window, ref. [50] found significant differences in the proportion of complex sentences produced by students in DLD and TD groups across the ages of 4 to 12 years old. Although the students with DLD made improvements with age, their performance remained lower than TD students, even until the age of 12 years [50].

### 1.4. Indices of Narrative Content and Its Relation to Disability

Narrative content has been primarily analyzed using the story grammar framework [51], or a holistic evaluation of the plot [52]. Although both have been used in the SLP literature, the bulk of the recent research relies on the story grammar approach due to its superior replicability [53,54,55,56]. Story grammar refers to key components of a story, including the sequence of events, and the episodic structure of a story [57]. The canonical elements include the character, setting, problem, plan, attempt, consequence, emotion, and ending. Although researchers have used a variety of methods when analyzing narrative samples, such as scoring rubrics and computerized software after the sample is segmented and coded [9], quantifying the inclusion and clarity of story grammar has not been fully automated. It generally requires a human to rate the extent to which elements are present in the sample and how understandable they are.

In a seminal study using the story grammar approach, ref. [58] compared the narratives of 40 students with and without DLD in 10-year-olds. Students in the DLD group retold significantly fewer story grammar elements compared to TD students, *F* (1, 38) = 7.71, *p* < 0.05. Students with DLD also retold fewer complete story episodes than the group without DLD, *t* (38) = 2.02, *p* < 0.05 [58]. Using a similar methodology, ref. [44] replicated the story elements findings in 7- to 10-year-olds with an effect size of *d* = 1.5, which indicated a large effect by itself and in relation to the literature [59].

Depending on the measure used, some studies report a ceiling effect. Ref. [60] examined a normative sample of 300 TD students and 77 students with DLD, ages 4 to 9 years, using the Edmonton Narrative Norms Instrument (ENNI) [61] for differences in story grammar. The inclusion of story elements was discriminated between the students for all age groups except at age nine. A ceiling effect, but with older children, was also shown in the study by [50] where story elements were distinguished between TD and children with DLD in age ranges of 4 to 6 years, 7 to 9 years, but not in the 10 to 12 years age range. The findings of significant differences up to nine years of age were also replicated in the study by [42], using a modified version of a story grammar rubric to score for elements and plot complexity called narrative quality. This body of research suggests that story grammar elements and episode complexity can be used to differentiate students with DLD from typically developing students in the lower primary grades, but that there are potential ceiling effects in later grades. 

### 1.5. Comparing Narrative Form and Narrative Content in Relation to Disability

Even when several of the studies examined narrative form and content differences between TD students and students with DLD, only one study directly compared the relative strengths in form and content; ref. [44] compared narratives generated by two different cohorts of students evenly classified into children with and without DLD: a group of 26 9-year-olds, and a group of 40 7-year-old students. Students with DLD from both groups displayed distinct patterns of strengths and weaknesses. Using measures from the Test of Narrative Language (TNL) [62], approximately 27 out of the 33 students with DLD produced stories with either strong content and reduced grammatical accuracy or the opposite pattern; the six remaining students showed a balanced profile (i.e., strong in both, or low in both). These findings indicate that students with DLD face challenges when it comes to producing either the content or the form aspects of narratives, or both [44]. This also suggests that there may not be a single index that predicts language disability reliably.

### 1.6. Current Study and Research Questions

Narrative language sample analysis is a sensitive and beneficial tool for identifying language disability [42,43,44,46,48,50]. The evidence, however, is inconsistent regarding which aspects of narratives are most important for distinguishing DLD from TD, and it suggests that children with DLD may have uneven narrative strengths, either producing stories with poor content but accurate grammar, or reduced grammatical accuracy with elaborated content [44]. 

From the literature reviewed here, one main difficulty of comparing results from different studies arises from each specific study’s design; while some studies compare groups of students with DLD vs. TD children [43], others compare subsets of language disability to TD students [42]. Moreover, other researchers compared a group of students with DLD to students with other neurodevelopmental disorders [50]. Some researchers specified whether the children have been matched for age and nonverbal IQ [44,46] or grade [42], but additional matching variables were not used due to the difficulty of maintaining a large enough number of participants, and this limitation affects the generalizability of research findings.

Indeed, ref. [42] acknowledge that their study’s comparison sample may have a minority bias that could be important in storytelling skills, in that African American children were overrepresented in one of the comparison groups. Indeed, beyond the age factor, there is some evidence that contextual factors shape the language development of children [63]. More specifically, there is evidence that some narrative content or form measures vary based on the mother’s education [64], and minority status [65,66,67,68]. Another child-level factor for which there are documented differences in narrative measures is gender [42]. It would be desirable to control for these issues together when examining narrative indices to better understand student differences.

A second issue that has an impact on how language use is measured, and therefore what results are obtained from the language samples, involves the procedural choices of language processing and analysis. While most researchers have employed an automated analysis of language samples, diversifying the measurement approaches for form and content could enhance the confidence of the results. All of the studies reviewed above come from language samples that were audio recorded, transcribed, and analyzed with computerized software for narrative form and analyzed by human judges for narrative content. Although computerized software may seem fast and efficient in analyzing the count-based indices (i.e., NDW) after a language sample is transcribed and segmented, some form measures still require human coding before the computer reads the transcription and generates a report (e.g., %GE and SI). Another approach to analyzing narrative samples is through the use of scoring rubrics, where the sample is transcribed, and human coders give scores according to the inclusion, clarity, or complexity of the rubric items [28]. The use of scoring rubrics can be applied to both content and form indices and has the advantage of real-time scoring (i.e., during story retell) or the delayed scoring of an audio recording of the sample. Without the necessity for transcription, this approach could be more feasible in practice. For research, however, transcribing before scoring is commonplace, even when it can be feasibly scored without it [69,70]. The question remains whether different coding/analysis approaches that vary in time and cost can yield comparable results. As educators and clinicians are extremely busy [71], it is prudent to consider utility vis-à-vis practicality. 

There is a need to thoroughly explore the various indices of students’ narrative language so that a precise set of indicators of DLD can be identified. It is important to increase the rigor with which this research is done, which may involve a larger number of matching variables and the comparison of different approaches to measuring the same index with varying efficiency. This would enable clinicians to select a measure or a set of measures with the greatest predictive power while also minimizing the time and effort needed to collect and score the sample. Therefore, in this study, we examined the relative utility of several content and form measures of narrative language produced by K-3rd grade students with and without disabilities, while controlling for child factors, including age, grade, and gender, as well as social factors, such as the mother’s education and ethnicity. The primary purpose of using several narrative language indices was to identify which of the various measures predicted language disability best when controlling for other language-related factors. The following research questions were addressed:

1. To what extent do narrative language form (i.e., %GE, NDW, SI, and Sentence Complexity) and content (i.e., story grammar) indices significantly differentiate students with disabilities from students with typical language development?
2. To what extent do narrative language form (i.e., %GE, NDW, SI, and Sentence Complexity) and content (i.e., story grammar) indices significantly predict language ability in K-3rd grade students?
3. What differences exist across grades in terms of narrative language form (i.e., %GE, NDW, SI, and Sentence Complexity) and content (i.e., story grammar)?

In light of the above, the study’s primary objectives are to determine the extent to which narrative language form (i.e., %GE, NDW, SI, and Sentence Complexity) and content (i.e., story grammar) indices significantly differentiate students with disabilities from students with typical language development, to determine the extent to which narrative language form (i.e., %GE, NDW, SI, and Sentence Complexity) and content (i.e., story grammar) indices significantly predict language ability in K-3rd grade students, and to examine the group differences across grades in terms of narrative language form (i.e., %GE, NDW, SI, and Sentence Complexity) and content (i.e., story grammar).

## 2. Materials and Methods

### 2.1. Participants

The data used in this study were drawn from a larger study with 1037 racially, ethnically, and economically diverse K-3rd grade students [72]. Participants were recruited from 60 before- and after-school care and summer programs coordinated by the school district, the county, or the city’s parks and recreation over a 15-month period. Consent for participation was obtained by Research Assistants (RAs) visiting each site during student pick-up times. After the consent form was signed, caregivers completed a short demographic survey composed of questions regarding the student’s race, ethnicity, spoken language(s), and special education status. Project identification numbers were used when documenting students’ data to ensure anonymity. Language samples produced by English monolingual students (*n* = 50) whose parents reported that they had an Individualized Educational Plan (IEP) and reported concerns about their language were included in this study. Fifty TD students with no IEP or language concerns as reported by their parents were matched to the students with disabilities on age, gender, grade, their mother’s education, and ethnicity using propensity score matching. It should be noted that while parents did not specifically report students’ diagnoses (e.g., DLD), we used the merging of an IEP and language concerns as a proxy for language-based disability. This was necessary because there is currently extreme variation in how students with language disabilities are classified and diagnosed in U.S. schools. Participant characteristics are displayed in Table 1.

### 2.2. Research Team 

The research team, who collected and scored the language samples, consisted of four full-time salaried RAs with undergraduate degrees and seven undergraduate student RAs. Check-out procedures were established to ensure that RAs mastered the language elicitation protocols prior to the study’s data collection. RAs completed an initial two-hour training introducing the procedures and scripted elicitation guides, and then engaged in extensive practice. RAs performed the elicitation procedures with repeated practice until 100% fidelity was achieved, then advanced to collecting language samples for the study. All RAs were trained in language transcription, but only four RAs, those who were full-time staff, were trained to segment the transcribed samples into communication units (c-units), and complete coding. Following the completion of training, RAs were given a set of practice samples to segment and code, then a follow-up session was scheduled to discuss the trainees’ scores and clarify scoring procedures. Additionally, coders participated in weekly calibration meetings to discuss difficult samples and review coding procedures. This was necessary to prevent scoring drift over time given the large number of samples that RAs coded.

### 2.3. Procedures

Language samples were collected in 10 to 15 min sessions. RAs elicited two narrative retell oral (NRO) samples and two narrative generation (NGO) samples within one session. To elicit the narrative samples, standardized materials and procedures were used (available for free at http://trinastoolbox.com/research_ALPS.html). These included photo stimuli, scripts for elicitation, and model stories for the retell tasks. There were nine total sets of photos, each set consisting of three photos depicting a problem, an attempt to solve the problem, and a resolution. Before eliciting the samples, RAs presented three different sets of three photos and asked the students to select the photo set they wanted to talk about. The ability to choose a familiar or preferred topic reduces the potential bias in language sampling procedures [73]. Once the child made a choice, the selected three photos were laid out in front of the child and the others were removed. The choice procedure occurred for each of the four samples elicited without the replacement of the previously selected photo sets. 

With the three photos displayed, RAs used the scripts to engage the students in retell or generation tasks. For the retell task, students listened to an age-appropriate story that corresponded with the three photos and then retold that story while the photos remained visible. For the generation task, students made up a story about the three photos displayed. All four narrative elicitations were audio recorded, and the students’ productions were transcribed and coded using multiple measurement approaches (see below). Because some students did not produce the intended four samples (two oral narrative retells and two narrative generations), we used mean scores from the number of samples each student produced. 

### 2.4. Transcription and Coding

The recorded language samples were transcribed in accordance with corpus linguistic standards and then scored using the Narrative Language Measures (NLM) Flowchart [56]. Moreover, all the elicited samples were transcribed in accordance with the SALT conventions [47]. After samples were transcribed, the RAs segmented the samples into C-units following the SALT manual guidelines. A C-unit is defined as an independent clause with its modifiers. A clause is defined as a statement containing a subject and a verb. Each C-unit had to have the letter C at the beginning to indicate the child’s response and a period at the end so that the SALT 20 software would detect the C-units. During the segmenting process, RAs corrected spelling and capitalization errors that transcribers may have missed. RAs then coded each C-unit for grammar accuracy (i.e., grammatical error per C-unit) and the subordination index (i.e., number of clauses in each C-unit), and added grammaticality [G] and subordination [SI-1] codes to the end of each c-unit (e.g., C “and then she went to the dentist” [SI-1] [G].).

### 2.5. Measurement Approaches

#### 2.5.1. Systematic Analysis of Language Transcripts

The SALT 20 [47] is a software program used to analyze transcribed language samples, segmented into communication units—an independent clause with its modifiers—and marked with SI or grammar codes. Once transcribed and coded, samples were uploaded or transferred into the SALT 20 software for further analysis and summarization. The SALT calculated the following form indices: NDW, SI, and %GE.

#### 2.5.2. The Narrative Language Measures (NLM) Flowchart 

The Narrative Language Measures (NLM) Flowchart [56] is a scoring rubric designed to efficiently assess the content (called Narrative Discourse) and form (called Sentence Complexity) of narrative language. The Narrative Discourse section of the NLM Flowchart contains seven items that are scored in a flowchart fashion (i.e., start at the top and move downward according to yes/no questions), yielding scores of 2–4 points each. The seven items include: character (0–3), setting (0–3), problem (0–4), plan/attempt (0–4), consequence (0–4), ending (0–2), and emotion (0–3). Language samples with combinations of clear and complete episodic elements (i.e., problem, plan/attempt, consequence, and ending) earned 2–8 bonus points, depending on how many episodic elements were included in the narrative sample. The Sentence Complexity section contained six items: relative pronouns, verb/noun modifiers, vocabulary/rhetoric, temporal ties, causal ties, and dialogue. With the exception of dialogue (0–2), all items are scored on a 0–3 point scale. Narrative Discourse total scores were the sum of the seven scored items and the episodic bonus points. The Sentence Complexity total score included the sum of scores from the six items. 

The NLM Flowchart, which is included in the suite of assessments called the CUBED-3 [56,69], has been used to score generated oral narrative samples [74] and written narrative samples [75,76] in relation to oral language intervention. In these previous studies, the oral language intervention, which focused primarily on story grammar, improved the Narrative Discourse scores of preschool, kindergarten, and first grade students. Sentence Complexity scores were less sensitive to this intervention because language form was not targeted in this intervention explicitly. As reported in the CUBED-3 manual [56], the NLM Flowchart Narrative Discourse section has strong interrater reliability (*M* = 80.5%, range = 68–95%; *k* = 0.67) and correlates significantly with a norm-referenced assessment of language (*r* = 0.26). Evidence for the Sentence Complexity section is also adequate with strong interrater reliability (*M* = 89.5%, range = 86–96%; *k* = 0.74) and small association with a norm-referenced assessment of language (*r* = 0.19).

### 2.6. Fidelity and Reliability

Procedural fidelity was documented for the elicitation and transcription of samples. Reliability was documented for transcription, segmenting, as well as SI and grammatical accuracy coding. Task-specific fidelity and reliability checklists were developed for each activity, with varying numbers of items. The fidelity procedures involved the comparison of the examiner’s actual elicitation or the transcriber’s actual transcription with the intended standardized procedures. The reliability procedures involved the comparison of the original and the second transcriber’s, segmenter’s, or coder’s performance for each task. A third individual reviewed the first and second transcriptions to document adherence to transcription procedures and to calculate the percent of agreement between the two, which was calculated using the number of agreements, divided by the number of agreements plus disagreements, multiplied by 100.

First, independent RAs listened to 26% random samples of all audio recordings while using checklists to document the fidelity across all four contexts. The mean fidelity of elicitation was 99% (range = 35–100%). To examine the transcription fidelity, 22.5% of samples were reviewed by independent RAs, and a mean fidelity score of 99.2% (range = 75–100%) was obtained. For the reliability of transcription, 22% were independently transcribed from the audio files and checked for word-by-word agreement. The mean percentage of agreement was 91.65% (range = 0–100%). For the segmenting, subordination coding, and percentage of grammatical accuracy, 74% of samples were coded by a second independent RA, resulting in mean percent agreements of 99.4% (range = 1–100%) for segmenting, 95.2% (range = 0–100%) for subordination coding, and 94.6% (range = 1–100%) for the percentage of grammatical accuracy coding. Language samples falling below 80% agreement underwent a reconciliation process, in which a third transcriber listened to the audio recording and used their best judgment to decide on a final transcription, segmentation, and coding. 

### 2.7. Data Analysis Plan

A SALT [47] report was generated for each sample that included the NDW, SI, and grammatical accuracy (% GE) in each C-unit to help answer the research questions. The NLM Flowchart scores and scores from the SALT report were entered into a SAS (Version 9.4) for statistical analysis.

Prior to answering the research questions, we matched participants with (*n =* 50) and without (*n =* 50) disabilities. To do so, we used a specific propensity score matching procedure named the Optimal Fixed Ratio Matching technique in SAS software (Version 9.4), which selects all matches simultaneously and without replacement, aiming to minimize the total absolute difference. The groups were matched on their age, gender, grade, mother’s education, and ethnicity. Following the matching procedure, we tested the significance of random effects for each site and students nested within each site using a “proc mixed” method to inform our data analytic modeling approach and verify the accuracy of the matching procedure. The descriptive statistics, including the correlations, were then examined by group, to ensure that the data were normally distributed.

To address the first research question, a proc mixed method was used to examine the extent to which the form indices of narrative language using SALT 20 (NDW, SI, and % GE), narrative form using the NLM Flowchart (Sentence Complexity), and narrative content using the NLM Flowchart (Narrative Discourse) differentiate students with disabilities from TD students in K-3rd grade students. We used a Mixed Effect analysis to test the significance of both fixed effects for the narrative indices and random effects of site and the residual on disability status. 

For research question two, a logistic regression analysis was performed to examine which of the narrative form (NDW, SI, % GE, Sentence Complexity) and narrative content (Narrative Discourse) measures is most predictive of language ability in K-3rd grade students. All the narrative form and content variables were entered into the model simultaneously. A second regression analysis with only the significant variables was performed to improve model fit and enhance the interpretation of the results. To better understand score distribution between groups, we further analyzed the significant predictive variables. This analysis involved using the 25th percentile score of the TD group as a reference point to assess the relative performance of students with disabilities. Using the 25th percentile score is a common statistical approach to establish a performance baseline and gain insights into score distribution [77].

For research question three, descriptive and visual analyses were conducted by inspecting the bar graphs of each variable across grade levels (K-3) to determine variations by grade. A descriptive analysis was used because the sample size of each grade by group cell was too small to use inferential analyses.

## 3. Results

The data were normally distributed, and the samples’ demographic characteristics are displayed in Table 1. The initial test of the random effects of site and students nested within each site resulted in a not positive definite G matrix, which means the variance-covariance matrix for the random effects could not be properly estimated. In the subsequent model with the random effects of site only, the results suggested that the intercept (SE) for site, 0.21(0.08), was statistically significant, *p* < 0.01. However, the results of the model that included the random effects of site when testing the between-group differences on the matching variables resulted in a not positive variance-covariance matrix. Therefore, the random effect of site was removed and the model was estimated again. This model indicated that the group of children with and without disabilities were equivalent on all matching variables, *F*s(1, 92) = 0.00 to 1.98 and *p*s = 0.16 to 0.97. In both groups, the highest number of participants were in second grade (18 students), followed by third grade (15 students), then first grade (10 students), and kindergarten (7 students). One interesting finding was that there were substantially fewer females with disabilities than males. The descriptive characteristics of the narrative indices are displayed in Table 2. Finally, the bivariate correlations were linear and in the expected directions (see Table 3).

### 3.1. Differentiation between Groups

For the first research question, the results of the model that included the random effects of site when investigating the between-group differences of the narrative measures resulted in a not positive variance-covariance matrix. Therefore, the random effect of site was removed and the model was estimated again. The Narrative Discourse was the only measure that significantly differentiated between the group of TD students and students with disabilities (*β* = −0.043, SE = 0.013, *p* = 0.002, 95% CI [−0.06, −0.016]), suggesting lower scores on Narrative Discourse are significantly associated with an increased likelihood of disability status. Among the remaining indices of Sentence Complexity, SI, %GE, and NDW, no statistically significant effects on disability were observed (see Table 4).

### 3.2. Prediction of Disability Status

For the second research question, all narrative measures were included in the prediction of disability status (i.e., Sentence Complexity, Narrative Discourse, %GE, SI, NDW). As shown in Table 5, the results of the logistic regression indicated that the group of measures predicted 18.48% of the variability in classifying students with disabilities. However, Narrative Discourse was the only statistically significant predictor of disability status. For every unit increase in Narrative Discourse, there was a 21% decrease in the odds of being classified as having a disability (OR = 0.79, 95% CI [0.68, 0.92], *p* = 0.002). This means that as Narrative Discourse scores increase, the likelihood of being diagnosed with a disability decreases, with all other factors being equal. The remaining predictors, Sentence Complexity, SI, and NDW were not significantly related to disability. In a second logistic regression analysis that only included Narrative Discourse, the statistically significant predictor showed the model’s predictions accounted for 14.45% of the variance in disability classification. Here, a one-point increase in Narrative Discourse was associated with a 14.8% decrease in the odds of being classified as having a disability (OR = 0.85, 95% CI [0.78, 0.93], *p* = 0.001).

**Figure 1:**
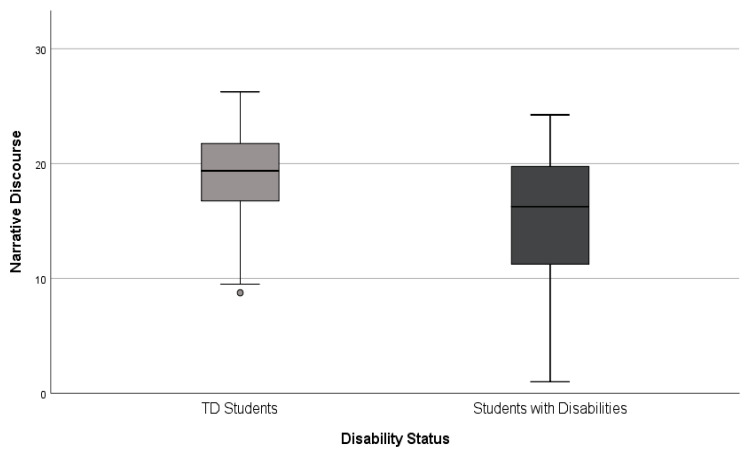
Narrative Discourse score distribution by disability group.

To further investigate the relationship between the performance of the two groups on the Narrative Discourse measure and their disability status, we examined the frequency distribution of students on this measure. There was a wide range of performances among students with disabilities, from very low (1) to very high (24) scores, which is reasonable given the students were drawn from four different grades. Although some students with disabilities had scores close to zero, none of the children in the TD student group performed that low. Although this analysis would have been more robust had we been able to examine the differences by grade, the grade-level sample sizes were too small. Therefore, these results should be considered exploratory only. Figure 1 illustrates this whole-group comparison.

### 3.3. Differences by Grades 

**Figure 2:**
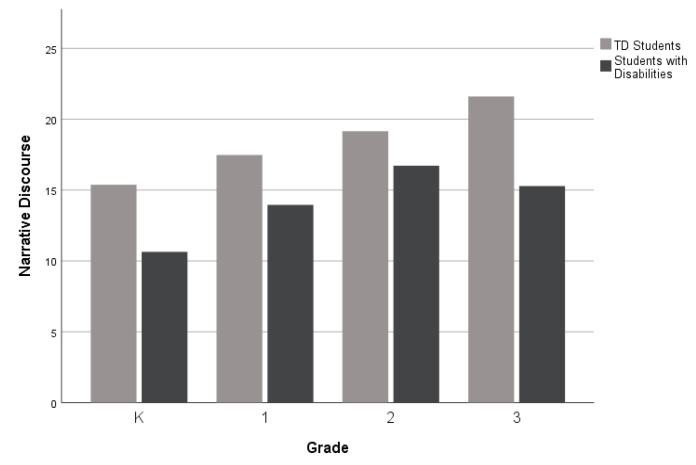
Mean Narrative Discourse scores by group and grade level.

**Figure 3:**
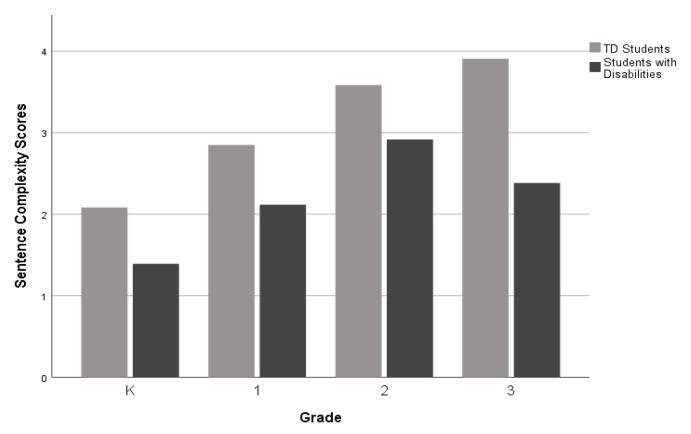
Mean Sentence Complexity scores by group and grade level.

**Figure 4:**
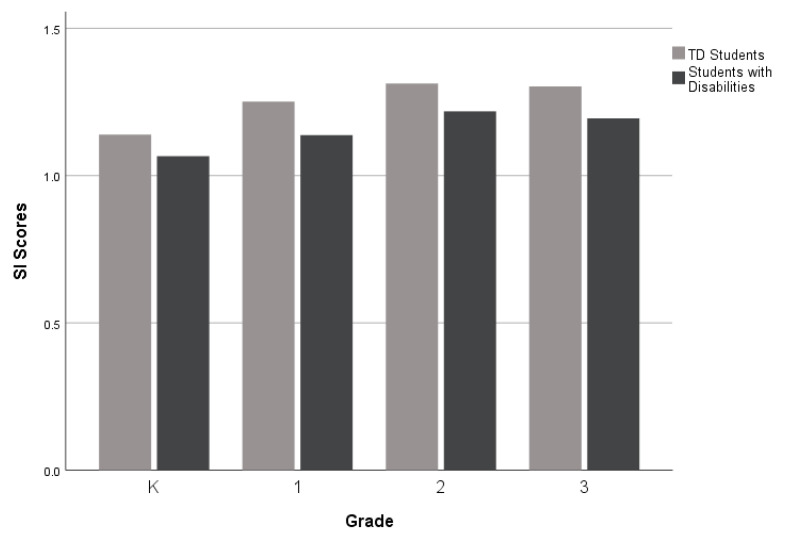
Mean Subordination Index scores by group and grade level.

**Figure 5:**
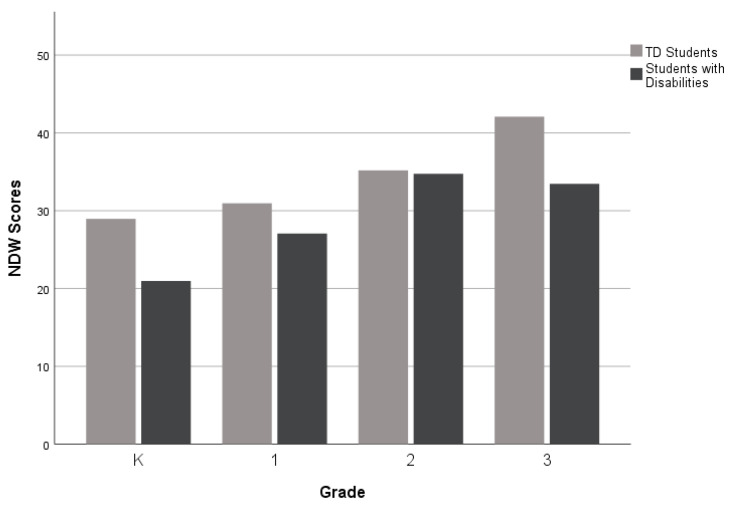
Mean Number of Different Words by group and grade level.

**Figure 6:**
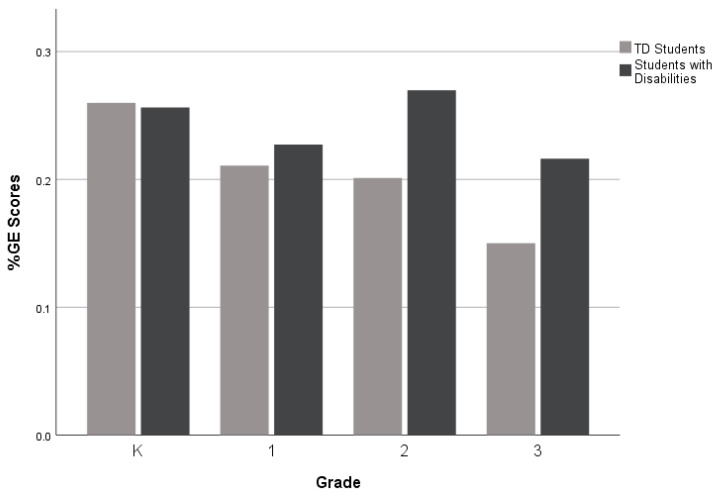
Mean % of Grammatical Errors by group and grade level.

For the third research question, which investigates narrative performance across different grade levels (K-3rd grade), as displayed in Figure 2, Figure 3 and Figure 4, there was a performance gap between the groups (TD students and students with disabilities) on all five measures. However, the gaps were wider for the Narrative Discourse and Sentence Complexity compared to the SI. The groups performed similarly on the NDW measure in 2nd grade (see Figure 5), but gaps between the groups appeared at the kindergarten, first, and third grades. Finally, as shown in Figure 6, TD students and students with disabilities performed similarly on the %GE in kindergarten and first grade, but gaps emerged in the second grade and remained consistent into the third grade. Collectively, the findings suggest that the most consistent difficulties across grades were documented in the Narrative Discourse and Sentence Complexity indices, with variation for the other indices depending on the grade. 

## 4. Discussion

The purpose of this study was threefold. First, we investigated the relative utility of five narrative measures in differentiating TD students from students with disabilities. Second, narrative language was used to predict disabilities among K-3 students. Third, we looked at variability in scores by disability status and grade. Previous studies emphasized the benefits of supplementing language evaluation with language samples, and several researchers have explored the extent to which language sampling differentiates and/or predicts language disability, e.g., [28,53,60,61]. Furthermore, the variability of language sampling and analysis methods in clinical practice warrants additional investigation to facilitate the selection of approaches among clinicians. The present study included the most common measures used in the literature to distinguish students with disabilities from TD students using a SALT, e.g., [42,44,46,50,61,62], and a scoring rubric for narrative discourse and sentence complexity (NLM Flowchart) [56]. To improve methodological accuracy and group comparisons in identifying students with disabilities, we considered important contextual factors like age, gender, maternal education, and ethnicity during the matching process. Moreover, we integrated the mean scores for the narrative retells and narrative generations to ensure consistency among participants, and the mean score of the four (or fewer) samples for each child was considered for analysis. 

### 4.1. Group Differences in Narrative Language Content and Form Measures

Our first research question focused on differentiating students with disabilities from students with typical language development as measured by their NDW, SI, %GE, Sentence Complexity, and Narrative Discourse. Most of the findings in the present study were consistent with prior work comparing oral narrative production in TD students and students with DLD (e.g., [46,50,61]). This body of research indicated that TD students, compared to students with disabilities, produced oral narratives with shorter and less complex sentences, fewer numbers of words, more grammatical errors, and fewer narrative discourse elements. Our findings supported the Narrative Discourse measure as the only variable that can distinguish between TD students and students with disabilities; there were no significant differences between groups in the NDW, SI, %GE, and Sentence Complexity. This finding is not consistent with previous literature (e.g., [46,48,61,78]). The reason for this discrepancy between our findings and the previous research could be due to sampling, elicitation, and/or methodological differences. First, the sample used in the previous research differed from the present study in that the former only included students with language impairment, whereas our study included students with various disabilities whose parents expressed language concerns. Second, elicitation differences could be another reason; the researchers in the previous studies [48,61,78] used story generation as the only method of narrative elicitation, while we elicited retell and generated narratives and used a combined metric in the analyses. Third, an important methodological difference is that the previous studies did not match samples for confounding factors like the mother’s education, and ethnicity, which are known to impact language and academic outcomes [79]. In our study, we controlled for these variables, increasing our confidence that group differences were not influenced by these factors, but rather by variations in language proficiency or the presence of disabilities. Therefore, the Narrative Discourse measure has the potential to inform clinical decision-making in the assessment and classification of individuals with disabilities.

### 4.2. Narrative Measures to Predict Disability Status in K-3rd Grade Students

The second research question examined the utility of narrative measures in the prediction of disability status. The Narrative Discourse of the NLM Flowchart was the only measure that significantly predicted disability status in our sample. In the research literature, oral narrative has been found to strongly predict academic achievement [5] and reading comprehension [80,81]. Our findings further support the predictive power of the Narrative Discourse measure in relation to disability status. 

By reaffirming the role of the narrative structure, this study reinforces its potential as a useful measure for identifying disability. The findings of this study provide additional evidence that students with disabilities have the capability to generate stories with well-developed discourse structures. However, even when students with disabilities have the potential to achieve high scores on Narrative Discourse, it is crucial to raise concerns when observing low scores as they may indicate significant language learning limitations. Low scores on the Narrative Discourse measure could signify the presence of language difficulties that warrant attention and intervention. At this point, though, what defines a low score needs to be determined at each grade level, which was beyond the scope of this project.

### 4.3. Group Differences on Narrative Measures by Grade 

The final research question focused on differences between TD students and students with disabilities on narrative measures across grades K-3, which was answered descriptively. Prior research (e.g., [42,46,48,61,78]) provided evidence of a performance gap in narrative language between TD students and students with DLD. The present findings also provide evidence of performance gaps on some of the narrative form and content measures, with overall increases in sentence length, complexity, and the inclusion of more narrative discourse elements as grades advanced. Grade-level differences are expected and have been documented by previous researchers [61,73]. 

Among the narrative measures examined by grade, gaps between TD students and students with disabilities were consistently observed for Narrative Discourse, Sentence Complexity, and SI. Notably, the gaps were more pronounced in Narrative Discourse and Sentence Complexity. Acknowledging that the patterns observed in the present study are from cross-sectional data, these results should be interpreted as preliminary. 

An interesting pattern emerging from the present study was that the performance gaps on the %GE between students with disabilities and TD students were more pronounced in the second and third grades. In contrast, the performance gap for the NDW was inconsistent, but widened in the third grade, converging with the existing literature. These findings are consistent with previous research demonstrating that students with DLD experience persistent challenges with grammatical accuracy, language complexity, and literate language [37,46,82]. The pattern also aligns with previous research (e.g., [42]) showing larger differences in older children. Ref. [42] compared the narrative performance of subgroups of students with DLD and TD students in kindergarten, second, and fourth grades, and found that there was a small gap in performance on narrative measures in second grade, but the gap increased by fourth grade. One possible explanation for this trend could be the increased linguistic demand as grades progress, which poses challenges for students with disabilities, making it difficult for them to keep pace with TD students. The results suggest that these measures are more sensitive in detecting disability at higher grade levels compared to lower grades, such as kindergarten and first grade. The present findings provide valuable insights into the specific stages and indices where performance differences between TD students and students with disabilities become evident. 

In summary, our study showed that the measures of Narrative Discourse and Sentence Complexity exhibit heightened sensitivity in differentiating TD students from those with disabilities across all grade levels within our sample. These results highlight the distinct value and utility of the Narrative Discourse and Sentence Complexity sections of the NLM Flowchart in identifying and distinguishing between these two student groups throughout the K-3rd grade levels, particularly with the gaps increasing in older children, as they face texts with greater linguistic demands.

### 4.4. Clinical Implications

This study revealed that the Narrative Discourse of the NLM Flowchart was the only measure that differentiated students with disabilities from TD students across grades and could predict disability status using narrative language samples. This implication is slightly tempered because our sample included students with various disabilities, not just students with DLD. Nonetheless, study findings suggest that producing language samples with a sufficient number and clarity of narrative discourse elements is a challenge for many students with IEP and parent-reported language concerns. Therefore, a Narrative Discourse measure may be useful for students with disabilities, including students with DLD. Finally, these findings speak to the importance of monitoring students’ Narrative Discourse performance and their use of complex sentences. If narrative language was evaluated routinely, it would be easier for educators to identify students who are struggling to learn the patterns of stories and who would benefit from intervention. 

The results of the present study do not support the use of SALT indices to identify students with disabilities. The Narrative Discourse was the only measure that differentiated and predicted disabilities in comparison to the SALT indices. The use of SALT requires specialized training and human coding, and is expensive. Although the use of the NLM Flowchart also requires training, it is substantially more intuitive than c-unit segmentation. The Narrative Discourse section of the NLM Flowchart is quicker and easier to score because it can be done in real-time. This eliminates the need for external resources or additional time, which is in short supply in elementary schools. Moreover, the NLM Flowchart is part of the CUBED-3 [56], which is free to download.

Using the Narrative Discourse measure from the NLM Flowchart in language evaluation could offer a comprehensive understanding of a child’s language use, encompassing critical elements such as story grammar, vocabulary, and pronouns. This wealth of information holds significant value for clinical decision-making, including uses in diagnosis, informing intervention, and monitoring progress over time. In addition to its ability to differentiate TD students from students with disabilities, the NLM Flowchart could potentially be used to inform instruction and intervention in the classroom. As narratives are mentioned throughout the primary grade academic learning standards [83], an assessment that yields information about students’ narrative language strengths and weaknesses can be a valuable classroom tool. 

### 4.5. Limitations and Future Direction

While this study provided valuable findings on content and form indices to differentiate students with disabilities from TD students, there were some limitations that should be considered. First, the study was limited in its sampling by including K-3rd grade students with disabilities with language concerns as reported by caregivers, not specific to students with language impairment, DLD, or other identified specific disabilities. In addition, we did not have a measure of disability severity. Second, the integration of mean scores for narrative retells and narrative generations was necessary to ensure consistency among participants as some students did not produce the expected number of samples. While this limitation does not detract from the present findings, future research is needed to replicate the results of this study with students with DLD and other identified diagnoses, and other age groups. 

Another limitation, which is considered above, is that the groups by grade patterns can only be considered preliminary because they are cross-sectional data. To draw a more robust conclusion and determine whether the observed patterns are not merely the result of group effects, longitudinal data with larger sample sizes are needed. Longitudinal data could also provide in-depth information regarding the relationship between narrative language and academic outcomes. Although this study informed that the Narrative Discourse was a predictor of disability status, we could not determine a specific cut score to indicate disability because the groups contained K-3rd grade students. A score of 18 is considered a high score for a kindergartener, but too low for a third-grader. Further research is needed with larger samples to examine the Narrative Discourse cut scores for each grade level.

Finally, this study only examined oral narrative language samples, which limits the generalizability of the findings to other types and modalities of language production. Therefore, the conclusions drawn from this study with regard to the utility of narrative measures in differentiating TD students from students with disabilities are limited to the oral modality. The NLM Flowchart has been used to score written narrative samples in intervention research [75,76], but more language sampling research in diverse narrative contexts is warranted. For example, research should investigate the differences in oral and written narrative language between students with DLD and TD students using the NLM Flowchart. This approach could provide valuable insights into the narrative profiles of students with disabilities and help identify potential performance patterns in both oral and written narratives. Clinicians could use students’ oral and written narrative abilities to identify students with language disabilities and inform comprehensive intervention strategies.

While it is important to consider the limitations of our study, they do not detract from the validity of our findings. By acknowledging these limitations, we maintain transparency and awareness of potential confounding factors. However, it is crucial to recognize that despite these limitations, our study offers valuable insight into measures that effectively differentiate TD students from students with disabilities. First, Narrative Discourse stood out as the only measure that differentiated students with disabilities from TD students. Second, Narrative Discourse emerged as the sole predictor of disability status. Third, in contrast to indices from SALT, the Narrative Discourse and Sentence Complexity were the two measures that consistently exhibited wide performance gaps between TD students and students with disabilities across the K-3rd grade levels. Given that narrative discourse elements can be explicitly taught and learned, as evidenced by a meta-analysis of 26 narrative interventions [23], our findings hold significant relevance for clinical decision-making and contribute to the existing knowledge in the field of language assessment and intervention for students with disabilities.

## Figures and Tables

**Table 1:** Demographic characteristics by group.

		Students with Disabilities (*n =* 50)		Students without Disabilities (*n =* 50)	
		M	SD	M	SD
	Age	7.34	1.17	7.38	1.14
	Grade	1.82	1.02	1.88	1.00
	Mother’s Education	1.82	1.02	1.88	1.00
		*n*	%	*n*	%
Gender/Sex					
	Female	9	18	9	18
	Male	41	82	41	82
Race/Ethnicity					
	African American	13	26	13	26
	Asian American	2	4	0	0
	Hispanic	12	28	12	28
	White	23	46	25	50

**Table 2:** Descriptive statistics for students with (*n =* 50) and without (*n =* 50) disabilities.

	Students with Disabilities				Students without Disabilities			
	M	SD	Skew	Kurtosis	M	SD	Skew	Kurtosis
Sentence Complexity	2.38	1.90	0.71	−0.40	3.36	1.87	0.70	1.01
Narrative Discourse	14.89	6.27	−0.57	−0.59	19.15	4.03	−0.64	0.12
%GE	0.24	0.19	0.95	0.22	0.19	0.15	1.53	2.60
SI	1.17	0.22	−0.98	1.76	1.28	0.18	0.99	1.99
NDW	31.50	15.25	0.16	−0.60	39.46	12.45	0.17	0.02

Note. %GE = Percentage of Grammatical Errors; SI = Subordination Index; NDW = number of different words.

**Table 3:** Correlations for the Narrative Language Indices by Group.

	1	2	3	4	5
1. Sentence Complexity	-	0.74 ***	−0.32 *	0.61 ***	0.84 ***
2. Narrative Discourse	0.54 ***	-	−0.26	0.57 ***	0.77 ***
3. %GE	−0.07	−0.33 *	-	−0.24	−0.19
4. SI	0.52 ***	0.20	0.10	-	0.51 ***
5. NDW	0.80 ***	0.70 ***	−0.11	0.42 **	-

Note. The results for students with disabilities (*n =* 50) are shown above the diagonal. The results for students without disabilities (*n =* 50) are below the diagonal. %GE = Percentage of Grammatical Errors; SI = Subordination Index; NDW = number of different words. *** *p* ≤ 0.0001, ** *p* = 0.001, * *p* ≤ 0.05.

**Table 4:** Fixed Effects for group differences on the narrative measures.

	B	SE				95% Confidence Interval	
			df	F Value	*p*	Lower Limit	Upper Limit
Sentence Complexity	−0.036	0.07	94	0.62	0.434	−0.129	0.056
Narrative Discourse	−0.043	0.01	94	10.21	0.001	−0.069	−0.016
% GE	0.022	0.29	94	0.01	0.939	−0.567	0.613
SI	−0.23	0.28	94	0.65	0.423	−0.802	0.339
NDW	0.013	0.007	94	3.03	0.085	−0.001	0.028

Note. %GE = Percentage of Grammatical Errors; SI = Subordination Index; NDW = number of different words; df = Degrees of Freedom.

**Table 5:** Results from logistic regression with narrative content and form indices predicting disability status.

	B	SE	R^2^	Odds Ratio with 95% Confidence Interval		
				Lower Limit	Odds Ratio	Upper Limit
Model 1: Narrative content and form			0.18			
Sentence Complexity	−0.17	0.23		0.53	0.84	1.31
Narrative Discourse	−0.23 **	0.07		0.68	0.79	0.92
% Grammatical Errors	−0.13	1.52		0.04	0.88	17.49
Subordination Index	−1.45	1.48		0.01	0.23	4.21
Number of Different Words	0.07	0.04		0.99	1.07	1.15
Model 2: Narrative content only			0.14			
Narrative Discourse	−0.16 ***	0.84		0.78	0.85	0.93

Note. ** *p ≤* 0.001; *** *p ≤* 0.0001.

## Data Availability

The dataset analyzed for the current study (will be) available at the LDbase.org repository. https://ldbase.org.
